# Antiproliferative, Antimicrobial and Antiviral Activity of β-Aryl-δ-iodo-γ-lactones, Their Effect on Cellular Oxidative Stress Markers and Biological Membranes

**DOI:** 10.3390/biom10121594

**Published:** 2020-11-24

**Authors:** Aleksandra Włoch, Dominika Stygar, Fouad Bahri, Barbara Bażanów, Piotr Kuropka, Elżbieta Chełmecka, Hanna Pruchnik, Witold Gładkowski

**Affiliations:** 1 Department of Physics and Biophysics, Wrocław University of Environmental and Life Sciences, C.K. Norwida 25, 50-375 Wrocław, Poland; hanna.pruchnik@upwr.edu.pl; 2 Department of Physiology in Zabrze, Medical University of Silesia, Poniatowskiego 15, 40-751 Katowice, Poland; dstygar@sum.edu.pl; 3 Laboratory of Microbiology and Plant Biology, University of Mostaganem, Mostaganem 27000, Algeria; bahrifouad13@gmail.com; 4 Department of Veterinary Microbiology, Wroclaw University of Environmental and Life Sciences, Norwida 31, 50-375 Wrocław, Poland; barbara.bazanow@upwr.edu.pl; 5 Department of Biostructure and Animal Physiology, Wrocław University of Environmental and Life Sciences, C.K. Norwida 31, 50-375 Wrocław, Poland; piotr.kuropka@upwr.edu.pl; 6 Department of Statistics, Department of Instrumental Analysis, Medical University of Silesia, Ostrogórska 30, 41-200 Sosnowiec, Poland; echelmecka@sum.edu.pl; 7 Department of Chemistry, Wrocław University of Environmental and Life Sciences, Norwida 25, 50-375 Wrocław, Poland

**Keywords:** iodolactones, antimicrobial activity, antitumor activity, cytotoxic activity, antiviral activity, oxidative stress markers, hemolytic activity, biophysics research

## Abstract

The aim of this work was the examination of biological activity of three selected racemic cis-β-aryl-δ-iodo-γ-lactones. Tested iodolactones differed in the structure of the aromatic fragment of molecule, bearing isopropyl (**1**), methyl (**2**), or no substituent (**3**) on the *para* position of the benzene ring. A broad spectrum of biological activity as antimicrobial, antiviral, antitumor, cytotoxic, antioxidant, and hemolytic activity was examined. All iodolactones showed bactericidal activity against *Proteus mirabilis*, and lactones **1**,**2** were active against *Bacillus cereus*. The highest cytotoxic activity towards HeLa and MCF7 cancer cell lines and NHDF normal cell line was found for lactone **1**. All assessed lactones significantly disrupted antioxidative/oxidative balance of the NHDF, and the most harmful effect was determined by lactone **1**. Contrary to lactone **1**, lactones **2** and **3** did not induce the hemolysis of erythrocytes after 48 h of incubation. The differences in activity of iodolactones **1**–**3** in biological tests may be explained by their different impact on physicochemical properties of membrane as the packing order in the hydrophilic area and fluidity of hydrocarbon chains. This was dependent on the presence and type of alkyl substituent. The highest effect on the membrane organization was observed for lactone **1** due to the presence of bulky isopropyl group on the benzene ring.

## 1. Introduction

Cancer is one of the most serious causes of human death worldwide. Despite the introduction of many new anticancer drugs on the pharmaceutical market, mortality is very high, reaching about 9.6 million deaths in 2018 according to the World Health Organization [1]. The main reason for the low effectiveness of cancer treatment is the limited bioavailability of chemotherapeutic agents as well as serious side effects. Therefore, it is justified to search for new highly specific anticancer drugs that do not exhibit cytotoxic activity towards normal cells.

One of the factors leading to cancer may be bacterial, viral, and fungal infections. For example, *Candida* infections can significantly increase the risk of hematologic malignancy, head and neck cancer, and oral cavity cancer [2]. Hepatitis C and/or B virus can induce hepatocellular carcinoma [3]. *Helicobacter pylori* caused gastric and extragastric cancer in humans and has been associated with adenocarcinoma in the distal stomach by its ability to cause severe inflammations. *Chlamydia pneumoniae* was found to contribute as one of the ethological factors of lung cancer. Moreover, infection of the urinary tract with *Staphylococcus aureus*, *Klebsiella spp.*, *Proteus mirabilis*, and *Escherichia coli* has been found to cause bladder cancer. While the exact mechanism by which bacterial infections induce cancer is not fully understood, it is believed that it may be due to the release of free radicals which further damage DNA and regulatory proteins [4,5,6].

A sudden, uncontrolled growth of a number of free radicals is called oxidative stress and is another important factor contributing to the cancer development as well as neurodegenerative and cardiovascular diseases. It occurs when the balance between the formation and the removal of an excess of free radicals from the body is disturbed which leads to the oxidation of cell components, followed by the damage of proteins, lipids, and nucleic acids and disorders of cell structure and function. These destructive changes on the cellular level disturb the functioning of the whole organism [7]. A common indicator of an oxidative stress is an increase of reactive oxygen species (ROS), as well as a decrease of the activity of antioxidant enzymes which may reduce an antioxidant defense [8]. Therefore, in order to clarify the mechanism of action for anticancer compounds, it is important to examine their effect on an antioxidative status of the cells.

Lactones are bioactive compounds that possess various interesting properties, e.g., antifeedant [9,10,11,12], antimicrobial [13,14,15,16,17], and cytostatic [18,19,20,21,22,23] properties. They found application in medicine, pharmacy, cosmetology, food, and agriculture. Lactones are also intercellular signaling molecules used by many bacteria to monitor their population density in quorum-sensing control of gene expression. These compounds interact also with various eukaryotic cells to modulate immune responses [24,25,26].

Natural and synthetic lactones with an aromatic ring showing high anti-tumor activity deserve special attention [23,27,28,29]. In our previous research, we synthesized a series of β-aryl-δ-iodo-γ-lactones differing in substituents at the benzene ring which exhibited cytotoxic activity against two selected cancer cell lines: Jurkat (human T-cell leukemia) and D-17 (canine osteosarcoma) [30]. The mechanism of this activity can be explained by a determination of interactions between antineoplastic compound and the cell membrane and its effect on the biophysical parameters of membranes. The knowledge of this research is an important tool for better understanding the therapeutic activity of drugs and their toxic effects, because these processes may be related to the effect of the antitumor drug on the membrane [31,32]. This can play a significant role also in the future design of new more active drugs and delivery systems as well as the identification of new membrane targets.

**Figure 1:**
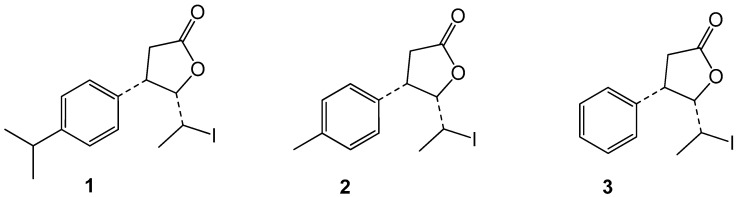
Chemical structures of tested β-aryl-δ-iodo-γ-lactones **1**–**3**.

The aim of this work was to extend biological assays for three previously synthesized β-aryl-δ-iodo-γ-lactones (Figure 1) by determination of antimicrobial, antiviral, antitumor, cytotoxic, antioxidant, and hemolytic activity as well as to study on the interactions of tested iodolactones with the protein–lipid membranes to better understand the mechanism of their biological activity.

## 2. Materials and Methods

### 2.1. Tested Compounds

Racemic iodolactones, namely *cis*-5-(1-iodoethyl)-4-(4′-isopropylphenyl)dihydrofuran-2-one (**1**), *cis*-5-(1-iodoethyl)-4-(4′-methylphenyl)dihydrofuran-2-one (**2**) and *cis*-5-(1-iodoethyl)-4-phenyldihydrofuran-2-one (**3**) were obtained in six-step syntheses from cuminaldehyde, 4′-methylbenzaldehyde, and benzaldehyde, respectively, at the Department of Chemistry, Wrocław University of the Environment and Life Sciences. The detailed synthetic procedures and characterizations of the compounds have been published elsewhere [30].

### 2.2. Antimicrobial Activity

#### 2.2.1. Microbial Strains

Bacterial strains (*Escherichia coli* ATCC 25922, *Pseudomonas aeruginosa* ATCC 27853, *Proteus mirabilis* ATCC 35659, *Bacillus cereus* ATCC 10876) and fungal strains (*Candida albicans* ATCC 10231 and *Aspergillus brasiliensis* ATCC 16404) were purchased from Pasteur Institute of Algiers (Algiers, Algeria).

#### 2.2.2. Disc Diffusion Method

The antimicrobial activity of synthetic lactones was assessed using the disc diffusion method [33]. Briefly, overnight, microbial cultures were adjusted to 0.5 MacFarland turbidity standards and spread on Petri dishes containing Mueller–Hinton and Sabouraud media for bacteria and fungi, respectively. Sterile paper discs (6 mm diameter) impregnated with solutions of lactone in DMSO were placed on the inoculated agar. Discs containing pure DMSO were used as negative control. Econazole and doxocycline were used as positive controls in the antifungal and antibacterial assays, respectively. The plates were incubated at 37 °C for 24 h for bacteria and at 25 °C for 48 h for yeast and 5 to 7 days for fungi. After incubation, the inhibition zone diameters were measured in millimeters (including disc diameter of 6 mm). The test was carried out in triplicates.

#### 2.2.3. Determination of Minimum Inhibitory and Minimum Bactericidal Concentrations

Minimum inhibitory concentration (MIC) and minimum bactericidal concentration (MBC) of the lactones were determined by the broth microdilution method [34,35]. The compounds were dissolved in DMSO and different concentrations ranging from 0.0156 to 2 mg/mL were prepared by serial two-fold dilutions in 96-well plates. A volume of 10 µL of bacterial inoculum was added to each well to achieve a final concentration of 1 × 10^4^ CFU/mL. One well containing only the medium and the inoculum was used as growth control. The plates were incubated at 37 °C for 24 h. MIC was defined as the lowest concentration of lactone showing no visible microbial growth.

MBCs were determined by serial sub-cultivation of 10 µL into microplates containing 100 µL of broth per well and further incubation at 37 °C for 24 h. MBC was defined as the lowest concentration of lactone showing complete absence of microbial growth.

### 2.3. Cytotoxic Properties

#### 2.3.1. Cell Lines and Media

The following lines were used in this experiment: NHDF (normal human dermal fibroblasts, PromoCell, C-12302), A549 (human lung carcinoma ATCC, No CCL-185 TM), HeLa (human cervix carcinoma ATCC, No CCL-2 TM), and MCF7 (human breast adenocarcinoma ATCC, No HTB-22 TM). They were obtained from American Type Culture Collection—ATCC (Rockville, MD, USA) and PromoCell GmbH (Heidelberg, Germany). Cell lines were cultured in Dulbecco’s Modified Eagle’s Medium—DMEM (Lonza, Basel, Switzerland). Media were supplemented with 10% fetal bovine serum (FBS) and 4 mM L-glutamine (Biological Industries, Kibbutz Beit-Haemek, Israel), 100 U/mL of penicillin and 100 µg/mL of streptomycin (Sigma-Aldrich, Munich, Germany).

#### 2.3.2. Cytotoxicity Assay

The studies of cytotoxic activity of lactones **1**–**3** were carried out according to EN 14476 [36]. Briefly, cell lines at a density of 4 × 10^4^ cells mL^−1^ were incubated in 96-well polystyrene plate (Nunc A/S, Roskilde, Denmark) for 24 h. Then, media from wells were removed. Product test solutions were prepared in 10% DMSO and DMEM supplemented with additional 10% FBS and L-glutamine. Solutions of lactones **1**–**3** at the concentrations from 10 mg/mL to 4.8828 μg/mL were prepared and transferred (100 μL) into cell culture units (wells of microtiter plates) containing monolayer of cells. Eight units were inoculated with each dilution. Plates were incubated in 37 °C and under 5% CO_2_ and observed daily for 4 days for the development of cytotoxic effect, using an inverted microscope (Olympus Corp., Hamburg Germany; Axio Observer, Carl Zeiss MicroImaging GmbH).

### 2.4. Antiviral Assay

Antiviral activity tests of iodolactones were conducted using human adenovirus 36 (Ad-36 virus—ATCC^®^ VR-1610™). Substances were tested using EN 14476 [36]. This standard describes a quantitative suspension test for the evaluation of virucidal activity in the medical area (Phase 2/Step 1), mixing one part by volume of test virus suspension (0.1 mL of 100TCID50 Ad-36 virus), one part by volume of interfering substance (0.1 mL of PBS), and eight parts by volume of disinfectant (iodolactones in concentration 10 mg/mL). At specified contact times (30 min), aliquots were taken, and serial dilutions up to 10^−8^ of each mixture were prepared. In eight repeats, 50 µL of each dilution was added to the microtiter plate containing a monolayer of confluent A549 cells. The plate was observed daily for up to 4 days for the development of viral cytopathic effect, using an inverted microscope (Olympus Corp., Hamburg, Germany; Axio Observer, Carl Zeiss MicroImaging GmbH). Then, residual infectivity was determined. According to PN-EN 14476:2005, a disinfectant is considered as having virucidal effectiveness if within the recommended exposure time the titer is reduced by ≥4 log10 steps (inactivation ≥ 99.99%).

### 2.5. Oxidative Stress Markers Analysis

NHDF cells, were incubated in in 6-well polystyrene plates (Nunc A/S, Roskilde, Denmark) for 24 h in two concentrations: the first was the starting concentration which was the same for all compounds (10 mg/mL), and the second was selected based on a cytotoxicity test (the lowest concentration which damaged cells), which was different for each particular compound. In the case of lactone **1**, the concentration was 0.078 mg/mL, for lactone **2** 0.156 mg/mL, and 0.3125 mg /mL for lactone **3**. Then cell culture medium was collected and frozen until testing. Cells were collected using EDTA trypsin and frozen again. The oxidative stress markers in cells homogenate was analyzed by determining superoxide dismutase analysis (SOD), catalase (CAT), glutathione peroxidase (GPx), glutathione-*S*-transferase (GST), the total antioxidant capacity (TAC), total oxidant status (TOS), and lipid peroxidation. All methods used in examination antioxidant status were described in detail in our previous papers [37,38].

For all oxidative stress markers, the data distribution of variables was evaluated by the Shapiro–Wilk test and quantile–quantile plot. The interval data were expressed as a mean value ± standard deviation in the case of a normal distribution. For the comparison of other factors (lactones **1**–**3** in different concentrations), the two-way parametric ANOVA with post-hoc contrast analysis was used. The homogeneity of variances was assessed by the Levene’s test. Statistical significance was set at a p value below 0.05, and all tests were two-tailed. Statistical analysis was performed using the Statistica 13.3 program (TIBCO Software Inc., Palo Alto, CA, USA, 2017).

#### 2.5.1. Catalase Activity (EC 1.11.1.6)

The catalase activity in cell homogenates was measured using Aebi methods. Briefly, 50 mM, pH 7.4 Tris/HCl buffer, and perhydrol were mixed with 50 μL of homogenate (Merck KGaA, Darmstadt, Germany). After 10 s, the absorbance was read every 30 s at λ = 240 nm for 2 min. The enzymatic activity was expressed in IU/mg protein where IU is the international unit of enzyme’s catalytic activity per mg of tissue [39].

#### 2.5.2. Glutathione Peroxidase (GPx) Activity (EC 1.11.1.9)

GPx activity was measured using the kinetic method [40], with *t*-butyl peroxide as a substrate. In this reaction, oxidized glutathione (GSSG) is regenerated in the presence of glutathione reductase (GR) and NADPH. GPx activity for all samples was expressed in IU/g protein where one international unit (IU) is the amount of µmol of NADPH oxidized in 1 min per 1 g of protein.

#### 2.5.3. Glutathione-*S*-Transferase (GST) Activity (EC 2.5.1.18)

GST activity was estimated, using the Habig and Jakoby kinetic method [41]. The reaction mixture containing reduced glutathione was added to the samples. After initial stabilization, 1-chloro-2,3-dinitrobenzene (in ethyl alcohol solution) was added, and absorbance changes were monitored using a Perkin Elmer Victor X3 reader at 340 nm wavelength for at least 3 min. GST activity was expressed as μmol of thioether formed within 1 min per 1 g of protein (IU/g protein).

#### 2.5.4. Superoxide Dismutase (SOD) Activity (EC 1.15.1.1)

Total SOD activity was measured, using the Oyanagui method [42]. In this method, xanthine oxidase catalyzes the production of superoxide anion that reacts with hydroxylamine to produce nitroso ion. The latter combined with *n*-(1-naphthyl)ethylenediamine and sulfanilic acid gives a colored combination that can be measured spectrophotometrically. Potassium cyanide (KCN) inhibits CuZnSOD activity; hence, CuZnSOD activity was assessed by calculating the difference between total SOD and MnSOD activity. Total SOD activity was presented as nitrite units (NU) per mg of protein. One NU is 50% blockage of nitrite ions formation as described by Oyanagui [42].

#### 2.5.5. Lipid Peroxidation

Lipid peroxidation was determined by malondialdehyde (MDA) concentration in test samples. MDA is the red product of reaction of lipid peroxides with thiobarbituric acid. MDA concentration was measured using the spectrofluorimetric method (wavelengths: 552 nm for emission and 515 nm for excitation; Perkin Elmer LS45 spectrofluorimeter (PerkinElmer, Inc., Waltham, MA, USA). MDA concentration was expressed in terms of μmol MDA/g protein and was calculated from the standard curve using 1,1,3,3-tetraethoxypropane (TEP) as an external standard. The original method was described elsewhere by Ohkawa et al. [43].

#### 2.5.6. Total Antioxidant Capacity (TAC)

TAC was measured using a commercial kit (Randox Co., County Antrim, UK). The 2.2′ azino-di-(3-ethylbenzothiazoline sulphonate) (ABTS) was incubated with a peroxidase (metmyoglobin) and hydrogen peroxide to produce the radical cation ABTS^+^, which has a relatively stable blue-green color and was measured at 600 nm. The suppression of the color was compared to the standard for TAC measurement assays (Trolox). The assay results are expressed as a Trolox equivalent (mmol/L). The inter- and intra-assay coefficients of variations (CV) were 1.1% and 3.8%, respectively.

#### 2.5.7. Total Oxidative Status (TOS)

The method according to Erel [44] uses the oxidation of iron (II) ions to iron (III) ions in an acidic medium. Then iron (III) ions with xylene orange form a colorful complex ranging up to a blue-purple coloration. Absorption readings were taken with a 560 nm filter on the Victor-X3 from Perkin Elmer. The TOS level was calculated from the calibration curve using H_2_O_2_ as the standard. Values are expressed in μmol/L.

### 2.6. Hemolytic Activity

Due to a simple structure and very important functions in the organism, red blood cells (RBCs) are a good model to assess toxicity of different compounds [45,46]. The hemolytic activity test was carried out using RBCs obtained from pig blood, because the percentage content of lipids is closest to the human erythrocyte. RBCs were obtained from examined and healthy pigs. The whole blood was taken to a physiological solution of sodium chloride with heparin added. Then the blood was centrifuged (2500 rpm/min) at 4 °C to remove the plasma and leucocytes and washed three times with cold phosphate-buffered saline isotonic solution.

The method applied was based on that described by Pruchnik et al. [45] with minor modifications. The following concentrations of each compound were used: 10, 20, 40, 60, 80, and 100 μM. All compounds were dissolved in ethanol. The control sample contained only ethanol in the same amounts as the samples tested. A final hematocrit in test samples was 1.2%. Samples thus prepared were incubated at 37 °C for 1 h, 24 h, and 48 h. After each incubation the hemolytic activity of the compounds was determined on the basis of the ratio of the hemoglobin concentration released from RBCs in tested probes to hemoglobin concentration released from RBCs in samples with total hemolysis multiplied by 100%. The measurement was performed at 540 nm using a UV-Vis spectrophotometer (Specord 40, Analytik Jena, Germany).

### 2.7. Biophysics Research

For biophysical research (fluorimetric and Fourier Transform Infrared Spectroscopy (FTIR) methods), isolated red blood cells membranes (RBCMs) were used, which were prepared according to the procedure described in the previous paper [47]. The amount of RBCMs was constant and was determined on the basis of protein concentration, which was assayed using the Bradford method [48]. RBCs do not have internal organelles; therefore, they are good candidates to isolate membranes. Furthermore, they are an ideal cell system for studying drug–membrane interaction [45]. Probes for fluorimetric research, 6-dodecanoyl-2-dimethylaminonaphthalene (Laurdan) and 1,6-diphenyl-1,3,5-hexatriene (DPH), were purchased from Molecular Probes (Eugene, OR, USA).

#### 2.7.1. Fluorimetric Method

The method was described precisely in the previous paper [45]. Briefly, lactones **1**–**3** were dissolved in ethanol to obtain the following concentrations: 10, 20, 40, 60, 80, and 100 μM. After adding the appropriate volume of solution of tested lactone to the mixtures of isotonic phosphate buffer, RBCMs, and the fluorescent probes (Laurdan or DPH, 1 μM), the samples were incubated for 1 h at 37 °C. After that the measurements were carried out at 37 °C on a fluorimeter Cary Eclipse (Varian, San Diego, CA, USA) and performed in three independent experiments. General polarization (GP) was calculated based on fluorescence intensity at the emission wavelength 440 nm and 490 nm of Laurdan probes. Fluorescence anisotropy (A) was determined based on fluorescence intensity of DPH probe.

The Statistica 13.0 was used for all statistical calculations. The results were analyzed by one-way ANOVA followed by Duncan test. Data are shown as mean values ± standard deviation (SD). *p* values < 0.05 were considered statistically significant.

#### 2.7.2. Fourier Transform Infrared Spectroscopy

The procedure has been described in our previous paper [49]. Briefly, the control (RBCMs with ethanol) and tested samples (RBCMs with 100 μM ethanolic solution of lactone) were applied at the ZnSn crystal, and the spectrum of each sample was taken at 37 °C using a Thermo Nicolet 6700 MCT (Thermo Fisher Scientific, Waltham, MA, USA). After that, the samples were incubated at 37 °C for 24 h to obtain a dry film (lipid–protein film) on the crystal and the measurements were repeated. Each single spectrum was obtained from 128 records at 2 cm^−1^ resolution in the range of 700–4000 cm^−1^. Preliminary elaboration of the spectrum was done using the EZ OMNIC v 8.0 program.

## 3. Results and Discussion

The subject of biological assays were three racemic cis-β-aryl-δ-iodo-γ-lactones, synthesized previously from corresponding aromatic aldehydes [30]. They differed in the structure of the aromatic fragment of molecule, bearing isopropyl (**1**), methyl (**2**), or no substituent on the para position of the benzene ring (**3**) (Figure 1).

### 3.1. Antimicrobial Assays

The antimicrobial activity of tested lactones was firstly evaluated using the disc diffusion method. The results indicated that lactones **1**–**3** did not show any antifungal activity which was in some contrast to our previous investigations in which lactones **1** and **2** inhibited up to 50% of the mycelium growth of four *Fusarium* strains (*F.culmorum*, *F.avenaceum*, *F.oxysporum*, and *F.solani*) [15].

Contrarily, tested iodolactones were active against two bacterial strains. The growth of *P.mirabilis* was inhibited by all tested compounds. The inhibitory zones were 12.5 mm ± 0.71, 10 mm ± 0.01, and 15 mm ± 0.5 for lactone **1**, lactone **2**, and lactone **3**, respectively, which were lower than for doxocycline used as a positive control (19 mm ± 1.41). The growth of *B.cereus* was inhibited to a lesser extent only by lactones **1** and **2,** with 9 mm ± 0.58 and 7 mm ± 0.01 zones, respectively, which was also lower than that determined for doxocycline (19 mm ± 1.41).

For the strains showing sensitivity in the disc diffusion method, the values of MIC, MBC, and MBC/MIC ratio were calculated (Table 1). According to Krishnan et al. [50], the compound is considered a bactericidal agent when MBC/MIC ratio ≤ 4 and a bacteriostatic agent when MBC/MIC ratio > 4. Based on the obtained results, we can conclude that the tested lactones possess a bactericidal activity. The highest activity was found for lactone **1** against *P.mirabilis*.

The particularly high sensitivity of *P.mirabilis* to the tested lactones is noteworthy because Gram-negative bacteria are generally more resistant to antimicrobials due to the outer membrane composed of chains of lipopolysaccharides. This layer forms a hydrophilic permeability barrier against the diffusion of hydrophobic antimicrobial compounds [51]. It would appear that the mode of action of lactones **1**–**3** towards Gram-negative bacteria is to make the cytoplasmic membrane permeable, by disintegrating the outer membrane and consequently releasing lipopolysaccharides. These can also deactivate essential enzymes and disrupt the functionality of genetic material, energy production, and the synthesis of structural components.

### 3.2. Cytotoxicity towards Normal and Tumor Cells

Cytotoxicity of tested lactones was evaluated in vitro on NHDF normal cell line and A549, HeLa, and MCF7 cancer cell lines. The results, shown in Table 2, are presented as minimal toxic concentration (MTC) which is defined as the minimal concentration of the compound that damages cells after 24 h of incubation.

The toxicity of tested lactones towards NHDF line increased with the enlargement of substituent size at C-4. The highest toxicity was observed for lactone with isopropyl group (**1**) followed by lactone with methyl group (**2**) and lactone with unsubstituted phenyl ring (**3**). The same order of activity was observed towards MCF7 line, but the toxicity levels of lactones **1** and **2** were higher compared with NHDF line. On the other hand, lactone **2** was more active towards A549 line and equally high activity of lactones **1** and **2** were found towards HeLa line. As with normal line, the least toxic against all cancer cell lines was lactone **3**. All lines were more sensitive to cisplatin compared to tested iodolactones.

The results showed some differences in the toxicity levels for normal and neoplastic cells in the case of lactones **1** and **2**, and no difference for lactone **3**. In our previous work, we reported the antiproliferative activity of compounds **1**–**3** against selected cancer cell lines: human T-cell leukaemia (Jurkat) and canine osteosarcoma (D-17) [30]. A similar relationship between the activity and the structure of aromatic fragment of the molecule was found. The most active was lactone with 4-isopropylphenyl substituent (**1**), and the least activity was observed for unsubstituted lactone **3**.

Except from A549 line, iodolactones **1** and **2** showed higher toxicity to the tumor lines than to the normal cells. This discrepancy may be due to the different interactions of tested compounds with cancer and normal cell membranes resulting from differences in their structure as well as biophysical and functional properties. These differences can promote or inhibit activity and toxicity of compounds [31]. Therefore, they are crucial for the understanding the biological activity of the compounds and, consequently, their effect on the therapeutic target.

### 3.3. Antiviral Properties

In concentrations of lactones which did not destroy tissue culture, the cytopathic effect caused by viruses was observed. It means, lactones **1**–**3** do not destroy Ad-36 virus in the concentrations that were non-toxic for cells.

Although adenoviruses and studies on their inactivation have been known for years, there is no approved drug for human adenovirus infection. Currently, only two antiviral drugs, namely cidofovir and ribavirin, are being used in first-line adenovirus therapy. However, their use is strongly limited due to their high toxicity. Hence, there is an increasing demand for research into the virulence of newly synthesized or natural substances. Among the most promising chemicals, the metal complex [Co(NH_3_)_6_]Cl_3_ is listed. Although the lactones tested in this project did not show any virulence against adenovirus 36, the literature referenced showed only a 50% reduction of this virus by [Co(NH_3_)_6_]Cl_3_ [52]. This justifies the need of further studies on the antiviral compounds.

### 3.4. Oxidative Stress Markers Analysis

Selected enzymatic and non-enzymatic oxidative stress markers were measured in the NHDF cell line after exposition to lactones **1**, **2**, and **3**. The results are presented in Table 3 and Table 4 for two different concentrations: starting concentration (SC) and minimal toxic concentration (MTC) given in Table 2 for NHDF line.

All assessed lactones led to a significant increase in oxidative stress (OS) in the selected cell lines. The statistical analysis of antioxidative stress markers showed that activities of all enzymes, GST, CAT, GPx, Total SOD, MnSOD, and CuZnSOD, as well as the levels of non-enzymatic markers such as TAC, TOS, and MDA were equally and significantly influenced by the type of β-aryl-δ-iodo-γ-lactones used in the experiment as well as by the concentrations of lactones **1**–**3** and interaction between the type of compound and lactone concentration (Table 3). The most harmful to the oxidative cell systems of the cells was lactone **1**, which at both concentrations significantly reduced the level of TAC and TOS and increased the level of MDA and activity of GST, CAT, GPx, and all isoforms of SOD in comparison to control (Table 3). Among three studied β-aryl-δ-iodo-γ-lactones, the lactone with no substituent at the phenyl ring (**3**) showed the least impact on the generation of OS in comparison to control. There was no significant influence of the concentration of lactone **3** on the MnSOD activity (Table 3 and Table 4). GST activity was significantly reduced by lactones **1**–**3** at MTC but not at starting concentration (Table 4). Lactones **1** and **2** had a similar impact on GST activity at MTC (Table 4, *p* = 0.337), whereas lactones **2** and **3** influenced the TOS level at SC. Using lactones **1** and **2** at their MTC led to the comparable reduction of deleterious impact of those compounds on TOS (Table 3 and Table 4).

Our results are in the line with other findings, which report a significant increase in oxidative stress parameters in the cancer type of the cell line after exposition to the several groups of lactones [53]. Thomasz et al. also showed that IL-δ increased ROS production by about 30% as well as increased lipid peroxidation levels about 19% in the HT-29 colon cancer cells. That may explain increased induction of apoptosis process as a result of increased oxidative stress [54]. We can conclude that antioxidative/oxidative balance of the cells was strongly disrupted by all tested compounds. The disruption of the antioxidative system was related to the type of compound, its concentration, and relations between the type of compound and its concentrations. The most deleterious effect on the antioxidative stress markers was exhibited by lactone with 4-isopropylphenyl substituent (**1**) and the least effect was observed for lactone **3** with an unsubstituted phenyl ring.

### 3.5. Hemolytic Activity

**Figure 2:**
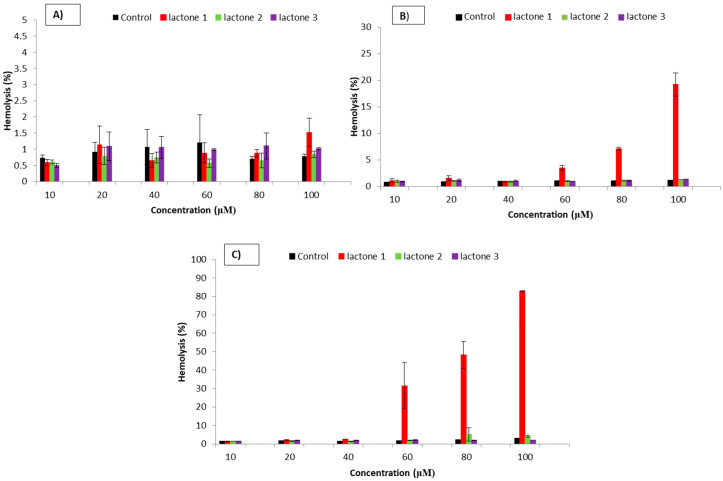
The dependence of hemolysis of red blood cells (RBCs) on the concentration of lactones **1**–**3** after: (**A**) 1 h, (**B**) 24 h, and (**C**) 48 h of incubation. The results are shown as mean values ± standard deviation (SD).

The degree of cytotoxicity of the compounds was evaluated on the basis of hemolytic activity by measurement of hemoglobin concentration released from damage RBCs. The results indicated that lactone **2** and lactone **3** did not induce the hemolysis of RBCs in the tested range of concentrations (from 10 to 100 μM) up to 48 h of incubation (Figure 2). The percentage of RBCs hemolysis modified with these compounds was at the control level and did not change significantly during the incubation. On the contrary, in the case of lactone **1**, the onset of hemolysis of erythrocytes was observed after 24 h of incubation at a concentration of 60 μM. At a concentration of 100 μM, the percentage of RBCs hemolysis reached 20%. Similarly, after 48 h of incubation with lactone **1** at 60 μM, a significant increase in RBCs damage was observed that at a concentration of 100 μM increased to almost 90%.

According to the toxicity classification of compounds, lactones with methyl (**2**) or no substituent (**3**) on the benzene ring are nontoxic, unlike lactone with isopropyl group (**1**) which is toxic at the highest concentration after 48 h of incubation [46]. A nontoxic effect on RBCs was also found earlier for the bromolactones with a 2,5-dimethylphenyl substituent and halogen derivatives of γ-lactones with a cyclohexane ring [23,49].

Different hemolytic activity of the tested compounds is probably due to the varying hydrophobicity and size of the substituent at the benzene ring affecting the interactions with the membranes. Lactone **1**, with higher hydrophobicity and a bulky substituent, penetrates the lipid bilayer deeper than lactones **2** and **3**.

### 3.6. Biophysical Research

#### 3.6.1. Fluorimetric Method

**Figure 3:**
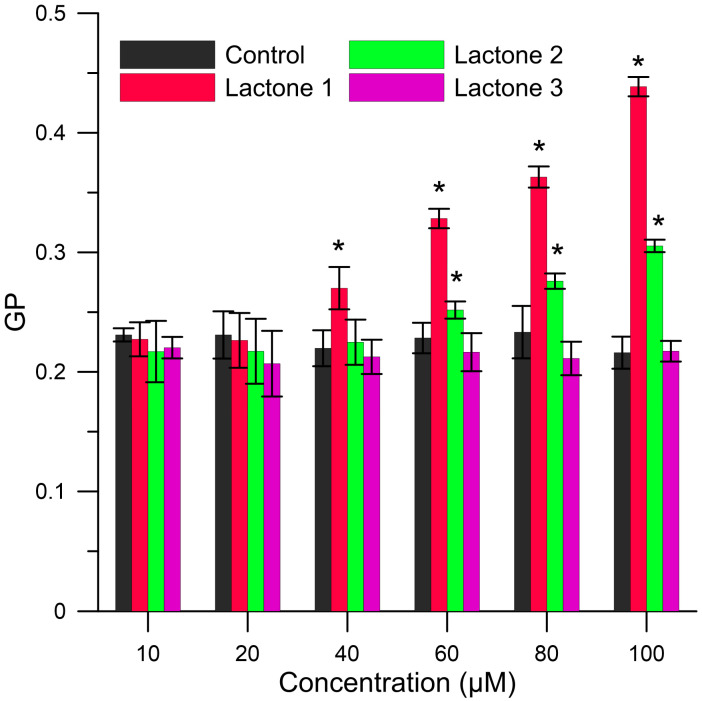
Values of generalized polarization (GP) of Laurdan probe for the red blood cells membranes (RBCMs; control) and RBCMs with addition of lactones **1**–**3** at 37 °C. Values are mean ± Standard Error of the Mean (SEM). Means labeled with asterisk (*) are significantly (*p* < 0.05) different from control.

The fluorimetric method was employed to monitor the effect of iodolactones on physicochemical properties of RBCMs like the hydration, stiffness, and packing of membrane building molecules. The monitoring of different areas in the membrane is possible using fluorescent probes. For this research, were used two fluorescent probes: Laurdan and DPH. Laurdan is incorporated into the apolar interior of the bilayer through hydrocarbon chains but fluorescent group is located at the level of lipid ester groups [55]. This fluorescent probe is sensitive to polarity modifications and reflects changes in hydration and packing order in the head group of lipids in RBCMs [55]. These changes were determined on the basis of generalized polarization (GP). Figure 3 shows the values of generalized polarization (GP) for pure membrane (control) and membrane modified with the compounds in six concentration at 37 °C. The results indicate an increase in GP values in the presence of lactones **1** and **2**. The large changes were observed for lactone **1** at the highest concentration accounting for approximately 100% of the control. In the presence of lactone **2**, the changes achieved at the same concentration reached 40% compared to the control. These changes were statistically significant (*p* < 0.05). The value of GP for lactone **3** was the same as for the control. An increase in the value of GP indicates a decrease in water content in the area of glycerol with a corresponding increase in the packing order in hydrophilic regions of the phospholipid bilayers.

**Figure 4:**
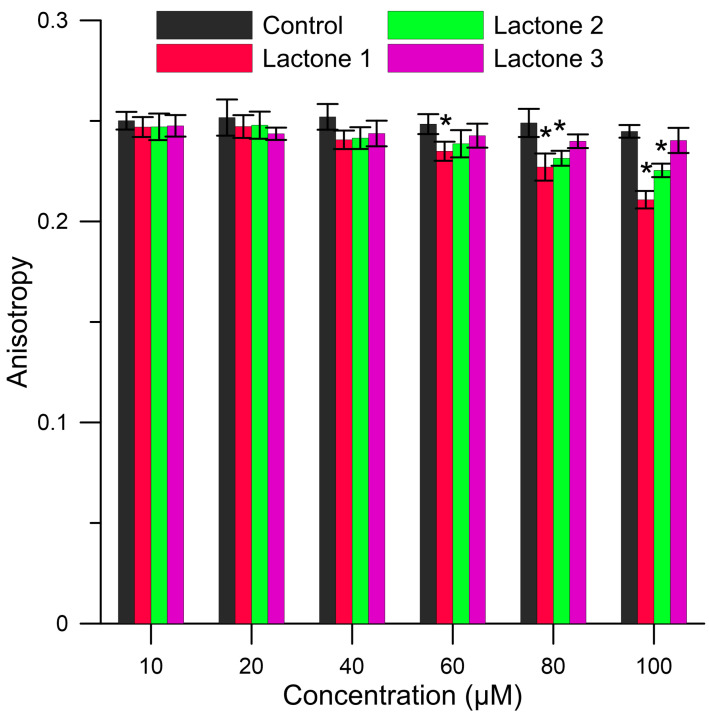
Values of anisotropy (A) of 1,6-diphenyl-1,3,5-hexatriene (DPH) probe for red blood cells membranes (RBCMs; control) and RBCMs with addition of lactones **1**–**3** at 37 °C. Values are mean ± Standard Error of the Mean (SEM). Means labeled with asterisk (*) are significantly (*p* < 0.05) different from control.

DPH is located in the hydrocarbon chains in the phospholipid bilayers and provides information about fluidity and rigidity of the local fluorophore environment based on the changes in fluorescence anisotropy (A). Changes of values of anisotropy for RBCMs (control) and RBCMs with addition of compounds are presented in Figure 4. The results show that the presence of lactones **1** and **2** caused a decrease of the value of anisotropy compared to the control sample. A decrease in the anisotropy was at the level of 14% and 8% for compounds **1** and **2**, respectively, and these differences were statistically significant (*p* < 0.05). The decrease in the value of anisotropy determines an increase of the membrane fluidity and a decrease of the hydrocarbon chains organization. This suggests that lactones **1** and **2** show an impact on the fluidity in the hydrophobic region of the lipid bilayers. However, the results also indicate that lactone **3** did not change the fluorescence anisotropy of the DPH probe in RBCMs in relation to the control sample (Figure 4) and did not affect the hydrophobic area of the membrane.

In summary, the results obtained from fluorimetric analysis showed that lactones **1** and **2** significantly interact with the membrane causing an increase of the packing order in the hydrophilic area as well as a slight increase in the fluidity in the area of hydrocarbon chains. Probably, these changes in membrane organizations are caused by the alkyl substituents on the para position of the benzene ring (hydrophobic substituents). Iodolactones **1** and **2** penetrate deeper into the bilayer, causing changes in the packing of the molecules which may weaken van der Waals interactions between hydrocarbon chains. These effects are more pronounced for lactone **1**, having a bulkier isopropyl group on the benzene ring than lactone **2**, which possesses a methyl group at the same position. Consequently, the lack of changes in biophysical parameters of membrane after the addition of lactone **3** is probably due to the lack of any alkyl substituent on the phenyl ring.

As shown by the above results, within the studied group of lactones, type and position of substituent on the benzene ring plays an important role in the interactions with the membrane. This finding can be also confirmed by our earlier studies of enantiomeric pairs of δ-bromo-γ-lactones and their δ-lactone analogs possessing a 2,5-dimethylphenyl substituent at the β-position of the lactone ring. They concentrated mainly in the hydrophilic part of erythrocyte membrane but had practically no influence on fluidity in the hydrophobic region. In that case, it was also found that enantiomers that showed higher interactions with the membrane exhibited higher antiproliferative activity [23].

#### 3.6.2. Fourier Transform Infrared Spectroscopy

**Figure 5:**
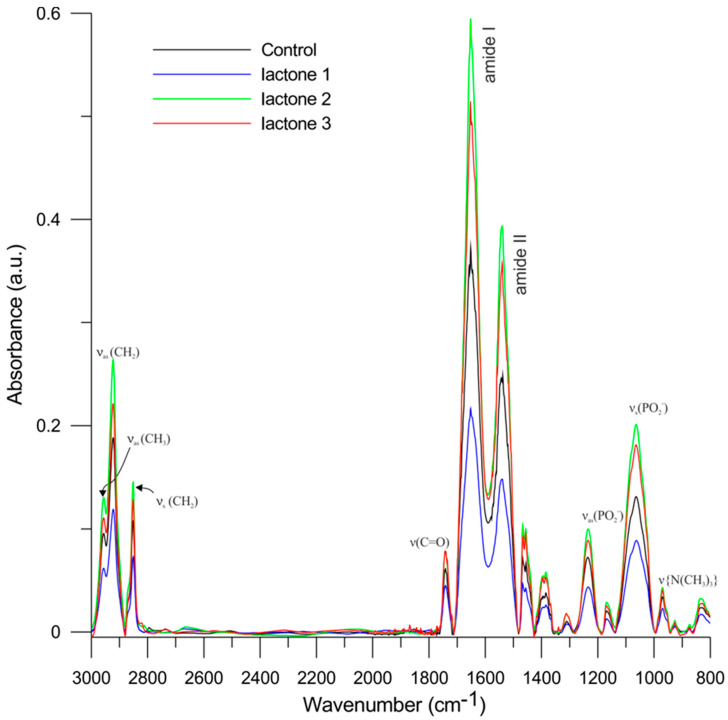
Fourier Transform Infrared (FTIR) spectra of red blood cells membranes (RBCMs, black lines) and of RBCMs modified with lactone **1**—blue lines, lactone **2**—green lines, and lactone **3**—red lines at 37 °C.

The FTIR method was employed to extend the information obtained in the flouorimetric research and to determine the effect of tested lactones on the main functional groups of the biological membrane. The most important bands of lipids considered in the RBCMs spectrum were band of hydrocarbon chains, carbonyl groups, phosphate group, and choline fragment. All bands with appropriate type of vibration are shown in Figure 5. The maxima of the main FTIR spectrum bands recorded at 37 °C for RBCMs and RBCMs in the presence of iodolactones are shown in Table 5.

In the hydrophobic region of the membrane, we considered bands located in the range of 3100–2800 cm^−1^ associated with lipids vibrations of CH_2_ (methylene) and CH_3_ (methyl) groups of alkyl chains. The maximum of asymmetrical and symmetrical stretching vibrations of the methylene groups are located at about 2920 cm^−1^ (*v*_as_CH_2_) and 2850 cm^−1^ (*v*_s_CH_2_), respectively. For the methyl groups, we considered only the maximum of asymmetrical stretching vibrations which is located at about 2956 cm^−1^ (*v*_as_CH_3_) [56]. Analysis of the IR spectra of hydrocarbon chains for RBCMs showed a small change in the frequency of signal of methylene group (asymmetrical and symmetrical stretching vibrations) only in the presence of lactone **1**. The maximum was shifted very slightly towards lower values of wave numbers compared to the control. The addition of lactones **2** and **3** did not change the signal frequency in this area. Based on these results, we can conclude that only lactone **1** causes a slight change in the ordering of hydrocarbon chains.

Another important functional group in the lipid molecules is the carbonyl group. The bands of the stretching vibrations of this group (*v*C=O) are located between the polar and non-polar parts of the membrane in the range of 1750–1700 cm^−1^. This group provides information on the degree of hydration in this region of the membrane [57,58,59]. The results did not show significant changes of frequency shift in this band, which indicates the lack of changes in the degree of hydration in this area of the membrane.

The hydrophilic area of the membrane of the erythrocyte is represented by the phosphate group and the choline fragment. Asymmetrical and symmetrical vibrations of phosphate group are located in the ranges of 1255–1225 cm^−1^ (*v*_as_(PO_2_^−^) and 1095–1085 cm^−1^ (*v*_s_(PO_2_^−^), respectively [60]. The asymmetric vibration bands are sensitive to changes in the polarity of the environment and the possibility of interaction with water by hydrogen bonds, whereas the symmetrical vibration band mainly reflects changes in the conformation of the phosphate fragment C-O-P-O-C [58]. The presence of iodolactones, particularly of lactone **1**, slightly shifted the maximum of asymmetric vibrations of the phosphate band towards lower values of wave numbers. In the case of symmetrical vibration, a similar situation was observed after addition of lactone **1** that caused a slight shift of this band in the same direction. Addition of the other two iodolactones caused a slight shift of the band in the opposite direction. The results indicated slight changes in the conformation of the phosphate group, especially after adding lactone **1** [49,61]. The phosphate band of the RBCMs summarizes absorption of infrared waves by different erythrocyte membrane phospholipids that occurs in all possible hydration states. An increase in the wavenumber of the maximum of this band indicates that the location of the lipid phosphate polar head group is in less polar environments and that the head group hydration decreased. In turn, a decrease in the wavenumber of the maximum of this band indicates that the lipid phosphate polar head group is located in more polar environments and that head group hydration increased [62]. Similar changes are also observed when the membrane embeds compounds having OH groups in their structure, which may also form hydrogen bonds with the phosphoric group lipids, e.g., polyphenols, plant extracts, carotenoids, complexes of platinum [61,63,64,65,66,67]. Therefore, the band located at 1235 cm^−1^ (for RBCMs) shifted toward lower frequencies in the presence of lactones probably indicates an interaction via hydrogen bonds between the PO_2_- group of the lipid and lactone **1** molecules. This suggests decreasing H-bonding between lipid molecules, which is indicative of the immobilization of this fragment of the lipid molecule, presumably due to a hydrogen bond formation. The obtained results confirm the conclusion stemming from the fluorimetric methods that iodolactones, in particular lactone **1**, influence the degree of ordering of the erythrocyte membrane lipid bilayer. Taking these results into account, we can confirm the interactions between the investigated compounds (especially lactone **1**) and the phosphate group of lipids. The most external part of the hydrophilic area is the choline fragment, and its characteristic band is located in three regions of the FTIR spectrum. The asymmetrical vibration (C–N^+^(CH_3_)) has the highest intensity, with a maximum at around 970 cm^−1^ [60]. The studies demonstrated that the lactones **2** and **3** shift the maximum of this band very slightly towards higher wavenumbers.

In the RBCMs spectra using FTIR methods, the main bands derived from proteins named as Amide I and II were also analyzed. Amide I is located in the range of 1610–1695 cm^−1^ and plays a key role in determining the type of protein secondary structure. Amide II occurs in the range of 1480–1575 cm^−1^ and is also used for determining the type of secondary structure [45,61,68]. These bands have the highest intensities throughout the RBCMs spectrum. The investigation showed that the addition of all tested compounds to the membrane caused only a slight shift of the maximum of the band Amide I and Amide II. The differences compared to the controls were higher for Amide II than for Amide I. The changes in the amide bands may suggest that the structure of proteins present in the membrane is slightly affected by tested iodolactones. Similar results were obtained for bicyclic δ-halo-γ-lactones with a cyclohexane ring [49].

The results obtained from FTIR suggest that all compounds affect the hydrophilic region of head groups of lipids by changing their packing order. However, lactone **1** had the highest effect on the membrane which is probably related to the presence of an isopropyl group on the benzene ring. The tested iodolactones only slightly affect the structure of proteins present in the membrane. The conclusions obtained from the FTIR method are very important due to the fact that the membrane components participate directly as messengers or regulators of signal transduction. In addition, protein–lipid interactions participate in the localization of signaling protein partners to specific membrane microdomains [69]. Therefore, affecting the physicochemical and conformational parameters of the membrane by the tested lactones can change cancer cell signaling.

## 4. Conclusions

The panel of biological assays carried out for testing *cis*-β-aryl-δ-iodo-γ-lactones showed that their activity is dependent on the presence and type of the alkyl substituent at the para position of benzene ring. In particular, the highest cytotoxic activity and the highest influence on the antioxidative stress markers was found for the lactone bearing isopropyl group (**1**), lower activity for lactone with methyl substituent (**2**), and the least activity for lactone **3**, possessing an unsubstituted phenyl ring.

The different activity of lactones with alkyl groups is probably the consequence of their different interactions with the cell membrane, which were particularly demonstrated in fluorimetric studies. The presence of a more bulky substituent at benzene ring results in deeper penetration of the lipid bilayer by lactone **1** and more significant changes in membrane organizations (increase of the packing order in the hydrophilic area, a slight increase in fluidity of hydrocarbon chains). The lack of significant changes in the biophysical parameters of the membrane after the addition of lactone **3** is probably due to the lack of an alkyl substituent.

In the light of these findings, further research is needed to understand the impact of the lactones on the membrane organization which is crucial to explain the mechanism of their biological activity.

## Figures and Tables

**Table 1:** Minimum Inhibitory Concentration (MIC) and Minimum Bactericidal Concentration (MBC) of lactones **1**–**3** (mg/mL).

Compound	*Proteus mirabilis* ATCC 35659			*Bacillus cereus* ATCC 10876		
	MIC	MBC	MBC/MIC	MIC	MBC	MBC/MIC
**Lactone 1**	0.25	1	4	0.5	1	2
**Lactone 2**	2	2	1	0.5	0.5	1
**Lactone 3**	1	2	2	-	-	-

ATCC: American Type Culture Collection; -: Not tested.

**Table 2:** The minimal toxic concentration (MTC) of lactones **1**–**3** against selected cell lines [mg/mL].

Compound	Cell Lines			
	NHDF	A549	HeLa	MCF7
**Lactone 1**	0.078	0.313	0.039	0.039
**Lactone 2**	0.156	0.156	0.039	0.078
**Lactone 3**	0.313	0.313	0.313	0.313
**Cisplatin**	0.005	0.01	0.005	0.01

**Table 3:** The levels of concentration assessed in the NHDF cells line after exposition to lactones **1**–**3**. The table shows results of the two-way ANOVA analysis between compound, concentration, and simultaneous influence of individual factors on selected oxidative stress markers. The results are shown as mean values ± standard deviation. SC—starting concentration, MTC—minimal toxic concentration. Statistical significance was set at *p* < 0.05. Statistically significant values are bolded.

		Compounds				
		SC	MTC	p Compound	p Concentration	p Interaction
Glutathione *S*-transferase (GST) (IU/g protein)	Control	3.6 ± 0.1	–	–	–	–
	Lactone **1**	83.2 ± 0.9	13.7 ± 1.2	**<0.001**	**<0.001**	**<0.001**
	Lactone **2**	24.2 ± 0.5	13.3 ± 0.4			
	Lactone **3**	11.0 ± 0.8	6.2 ± 0.9			
	Control	0.9 ± 0.1	–	–	–	–
Catalase CAT (IU/g protein)	Lactone **1**	152.1 ± 1.8	12.1 ± 0.1			
	Lactone **2**	12.0 ± 0.8	7.2 ± 1.4	**<0.001**	**<0.001**	**<0.001**
	Lactone **3**	5.6 ± 1.0	2.7 ± 0.3			
	Control	2.1 ± 0.2	–	–	–	–
GPx (IU/g protein)	Lactone **1**	154.9 ± 0.2	12.9 ± 1.0			
	Lactone **2**	77.3 ± 1.6	21.7 ± 0.2	**<0.001**	**<0.001**	**<0.001**
	Lactone **3**	11.2 ± 1.1	9.0 ± 1.1			
	Control	4.7 ± 0.1	–	–	–	–
Total SOD (NU/mg protein)	Lactone **1**	82.9 ± 0.1	16.4 ± 0.8			
	Lactone **2**	12.9 ± 0.2	4.6 ± 0.2	**<0.001**	**<0.001**	**<0.001**
	Lactone **3**	3.8 ± 0.5	2.6 ± 0.2			
	Control	4.3 ± 0.1	–	–	–	–
MnSOD (NU/mg protein)	Lactone **1**	52.9 ± 1.8	7.6 ± 0.9			
	Lactone **2**	8.3 ± 0.4	1.7 ± 0.6	**<0.001**	**<0.001**	**<0.001**
	Lactone **3**	1.2 ± 0.3	1.6 ± 0.2			
CuZnSOD (NU/mg protein)	Control	0.5 ± 0.1	–			
	Lactone **1**	30.0 ± 1.8	8.8 ± 1.0			
	Lactone **2**	4.5 ± 0.4	3.0 ± 0.5	**<0.001**	**<0.001**	**<0.001**
	Lactone **3**	2.6 ± 0.2	1.0 ± 0.1			
TAC (μmol/g protein)	Control	70.1 ± 0.1	–	–	–	–
	Lactone **1**	2.2 ± 0.1	36.0 ± 0.8	**<0.001**	**<0.001**	**<0.001**
	Lactone **2**	21.1 ± 0.6	32.5 ± 1.14			
	Lactone **3**	38.7 ± 1.4	44.7 ± 2.8			
TOS (μmol/g protein)	Control	31.6 ± 4.4	–	–	–	–
	Lactone **1**	0.5 ± 0.1	1.5 ± 0.1	**<0.001**	**<0.001**	**<0.001**
	Lactone **2**	1.7 ± 0.2	1.4 ± 0.1			
	Lactone **3**	1.7 ± 0.1	2.2 ± 0.3			
MDA (μmol/g protein)	Control	0.5 ± 0.1	–	–	–	–
	Lactone **1**	6.4 ± 0.1	2.6 ± 0.1	**<0.001**	**<0.001**	**<0.001**
	Lactone **2**	1.5 ± 0.1	1.7 ± 0.2			
	Lactone **3**	1.7 ± 0.1	1.0 ± 0.2			

glutathione S-transferase (GST), catalase (CAT), glutathione peroxidase (GPx), total superoxide dismutase (SOD), Mn-dependent superoxide dismutase (MnSOD), copper–zinc superoxide dismutase (CuZnSOD), total antioxidant capacity (TAC), total oxidant status (TOS), malondialdehyde (MDA).

**Table 4:** Multiple comparisons in contrast analysis. Post hoc analysis of selected oxidative stress markers in NHDF cells exposed to different concentrations of lactones **1**–**3** (SC—starting concentration, MTC—minimal toxic concentration). Statistical significance was set at *p* < 0.05. Statistically significant values are bolded.

	p_SC_ _vs. MTC_			SC			MTC		
	Lactone 1	Lactone 2	Lactone 3	p_1 vs. 2_	p_1 vs. 3_	p_2 vs. 3_	p_1 vs. 2_	p_1 vs. 3_	p_2 vs. 3_
GST (IU/g protein)	**<0.001**	**<0.001**	**<0.001**	**<0.001**	**<0.001**	**<0.001**	0.337	**<0.001**	**<0.001**
CAT (IU/g protein)	**<0.001**	**<0.001**	**<0.001**	**<0.001**	**<0.001**	**<0.001**	**<0.001**	**<0.001**	**<0.001**
GPx (IU/g protein)	**<0.001**	**<0.001**	**<0.001**	**<0.001**	**<0.001**	**<0.001**	**<0.001**	**<0.001**	**<0.001**
SOD (Nu/mg protein)	**<0.001**	**<0.001**	**<0.001**	**<0.001**	**<0.001**	**<0.001**	**<0.001**	**<0.001**	**<0.001**
MnSOD (NU/mg protein)	**<0.001**	**<0.001**	0.407	**<0.001**	**<0.001**	**<0.001**	**<0.001**	**<0.001**	0.872
CuZnSOD (NU/mg protein)	**<0.001**	**<0.01**	**<0.01**	**<0.001**	**<0.001**	**<0.001**	**<0.001**	**<0.001**	**<0.001**
TAC (μmol/g protein)	**<0.001**	**<0.001**	**<0.001**	**<0.001**	**<0.001**	**<0.001**	**<0.001**	**<0.001**	**<0.001**
TOS (μmol/g protein)	**<0.001**	**<0.001**	**<0.001**	**<0.001**	**<0.001**	0.955	0.502	**<0.001**	**<0.001**
MDA (μmol/g protein)	**<0.001**	**<0.01**	**<0.001**	**<0.001**	**<0.001**	**<0.05**	**<0.001**	**<0.001**	**<0.001**

p_SC vs. MTC_ for lactone **1**—SC values compared with MTC; p_SC vs. MTC_ for Lactone **2**—SC values compared with MTC; p_SC vs. MTC_ for Lactone **3**—SC values compared with MTC. Column SC shows comparisons between SC values for all three lactones. Column MTC shows comparisons between MTC values of all three lactones.

**Table 5:** Maxima of the main of FTIR spectrum bands for control (RBCMs) and tested samples (RBCMs + lactones).

Type of Bands	RBCMs	RBCMs + Lactone 1	RBCMs + Lactone 2	RBCMs + Lactone 3
*v*_as_(CH_2_)	2922.77	2922.14	2922.84	2922.95
*v*_s_(CH_2_)	2851.64	2851.28	2851.67	2851.68
*v*_as_(CH_3_)	2956.07	2956.25	2956.08	2956.18
*v*(C=O)	1741.67	1741.49	1741.77	1741.51
Amid I	1651.61	1651.96	1651.82	1652.75
Amid II	1538.01	1539.97	1539.16	1539.84
*v*_as_(PO_2_^−^)	1235.33	1233.93	1234.48	1234.70
*v*_s_(PO_2_^−^)	1063.47	1062.57	1063.70	1063.69
*v*_as_(C–N^+^(CH_3_))	969.88	969.85	970.14	970.18
